# Functional Lumen Imaging Probe (EndoFLIP) in Upper Gastrointestinal Disorders: Current Evidence, Clinical Applications, and Future Perspectives

**DOI:** 10.3390/diseases14080292

**Published:** 2026-08-13

**Authors:** Theodoros A. Voulgaris, Dimitrios I. Ziogas, Ioannis Stasinos, Eleni Koukoulioti, Eleni Koukouvaidou, Antonios Vezakis, Ioannis S. Papanikolaou

**Affiliations:** 1 Department of Endoscopy, Second Academic Surgical Unit, National and Kapodistrian University of Athens, Aretaieion Hospital, 11528 Athens, Greece; thvoulgaris@med.uoa.gr (T.A.V.); dimiziog95@gmail.com (D.I.Z.); avezakis@med.uoa.gr (A.V.); 2 Department of Gastroenterology, Athens Naval Hospital, 11521 Athens, Greece; 3 Department of Gastroenterology, 417 Army Equity Fund Hospital (NIMTS), 11528 Athens, Greece; ioannisstasinos@nhs.net; 4 Second Academic Department of Gastroenterology, Medical School, National and Kapodistrian University of Athens, 12462 Athens, Greece; ekoukoulioti@med.uoa.gr (E.K.); elinakoukouvaidou@gmail.com (E.K.)

**Keywords:** EndoFLIP, EndoFLIP achalasia, EndoFLIP POEM, pylorospasm gastroparesis, EndoFLIP G-POEM, EndoFLIP fundoplication

## Abstract

The Functional Lumen Imaging Probe (EndoFLIP^®^) system is an impedance-based technology providing real-time measurements of cross-sectional area (CSA) and intraballoon pressure across a luminal segment. Initially developed for esophagogastric junction (EGJ) evaluation in achalasia, the application of EndoFLIP has subsequently expanded to the assessment of both sphincteric and non-sphincteric regions throughout the gastrointestinal tract in a variety of clinical conditions, including eosinophilic esophagitis (EoE), post-fundoplication states, and gastroparesis. Its unique ability to be utilized in both the diagnostic and therapeutic settings, including the assessment of treatment response, makes it a valuable tool in the overall management of gastrointestinal motility disorders. The present review extends beyond a simple overview of EndoFLIP applications by providing a comprehensive evaluation of its performance in comparison with other diagnostic modalities, as well as the current knowledge gaps regarding its use. Study limitations and procedure-related aspects requiring further investigation are critically discussed, with the aim of supporting and guiding future research in this field.

## 1. Introduction

The Functional Lumen Imaging Probe (EndoFLIP) is a new modality launched during the previous decade trying to earn its place, mainly in the study of motility disorders of the upper gastrointestinal tract. It uses impedance planimetry within an inflatable, fluid-filled balloon to measure real-time cross-sectional area (CSA) and intraballoon pressure along a luminal segment [1]. It consists of a single-use flexible catheter with a distensible fluid-filled balloon containing a linear array of impedance ring electrodes and an intraballoon pressure sensor. During controlled volumetric distension, the console applies a low-amplitude current to the electrodes to measure local impedance, which is converted to CSA profiles along the balloon. Additionally, simultaneous pressure recording enables computation of the distensibility index (DI) (minimal CSA/pressure), pressure–volume curves, and compliance metrics displayed in real time to assess sphincter mechanics and lumen biomechanics [2].

Given the growing body of evidence supporting the use of EndoFLIP in the differential diagnosis of esophageal motility disorders, the Dallas Consensus recently proposed specific metrics and distension-induced contractility patterns to guide its use [3]. Currently, EndoFLIP is used as a complementary tool to manometry and imaging, aiding diagnosis and procedural assessment when it comes to esophageal and gastric motility disorders such as achalasia, post-fundoplication, or gastroparesis [4,5].

This narrative review aims to summarize the current evidence regarding the diagnostic and therapeutic applications of EndoFLIP in upper gastrointestinal disorders, with emphasis on clinically relevant metrics, proposed cut-off values, procedural guidance, and areas where evidence remains insufficient.

## 2. Sedation and Anesthesia Considerations

A longstanding point of debate has been whether sedation affects sphincter function and, consequently, influences EndoFLIP measurements. This issue is particularly important because there is currently no consensus regarding a standardized sedation protocol for motility studies, and sedation practices vary considerably across centers. While chronic opioid use is associated with esophageal dysfunction, the acute effects of these agents remain unclear. A recent systematic review suggested that remifentanil may reduce lower esophageal sphincter (LES) pressure, whereas fentanyl may increase it [6]. However, it should be noted that these effects were generally insufficient to alter motility diagnoses according to the Chicago Classification in most studies [6]. A recent retrospective study by Pezzino et al. [7] aimed to evaluate the impact of different sedation methods on EndoFLIP performance relative to high-resolution manometry (HRM). They included 454 patients, of whom 174 received conscious sedation with midazolam and fentanyl and 280 received monitored anesthesia care with propofol, reporting no differences in FLIP outcomes between the two sedation modalities (*p* = 0.0306 among all FLIP classifications). Another area of uncertainty is whether general anesthesia with inhalational anesthetics has a different effect on FLIP measurements compared with other sedation methods. In this setting, the abovementioned systematic review concluded that inhaled anesthetics (mainly halogens) may predispose to a reduction in LES pressure; however, only a limited number of studies have evaluated their effect [6]. These results were confirmed in a recent retrospective study of 49 patients undergoing peroral endoscopic myotomy (POEM) who received either intravenous anesthesia with propofol or inhalational general anesthesia with sevoflurane [8]. The authors observed a significant reduction in esophageal pressure among patients receiving sevoflurane-based anesthesia [8]. Taken together, the available evidence indicates that sedation with opioids or propofol has no clinically meaningful impact on FLIP measurements when administered at standard doses. Regarding general anesthesia with inhaled agents, further studies are needed to better define its potential effects.

## 3. Esophageal Motility Disorders

### 3.1. Esophagogastric Junction (EGJ) Evaluation

In a pivotal study in 2013, Pandolfino et al. [9] evaluated the use of EndoFLIP in patients with achalasia (treated and untreated) as well as in controls. The authors came to the conclusion that FLIP provided a useful measure of EGJ distensibility in achalasia patients that correlated with symptom severity. Additionally, they were the first to propose a DI cut-off of 2.8 by using receiver operating characteristic (ROC) analysis for diagnosing clinically significant obstruction at the level of the EGJ [9].

**Figure 1:**
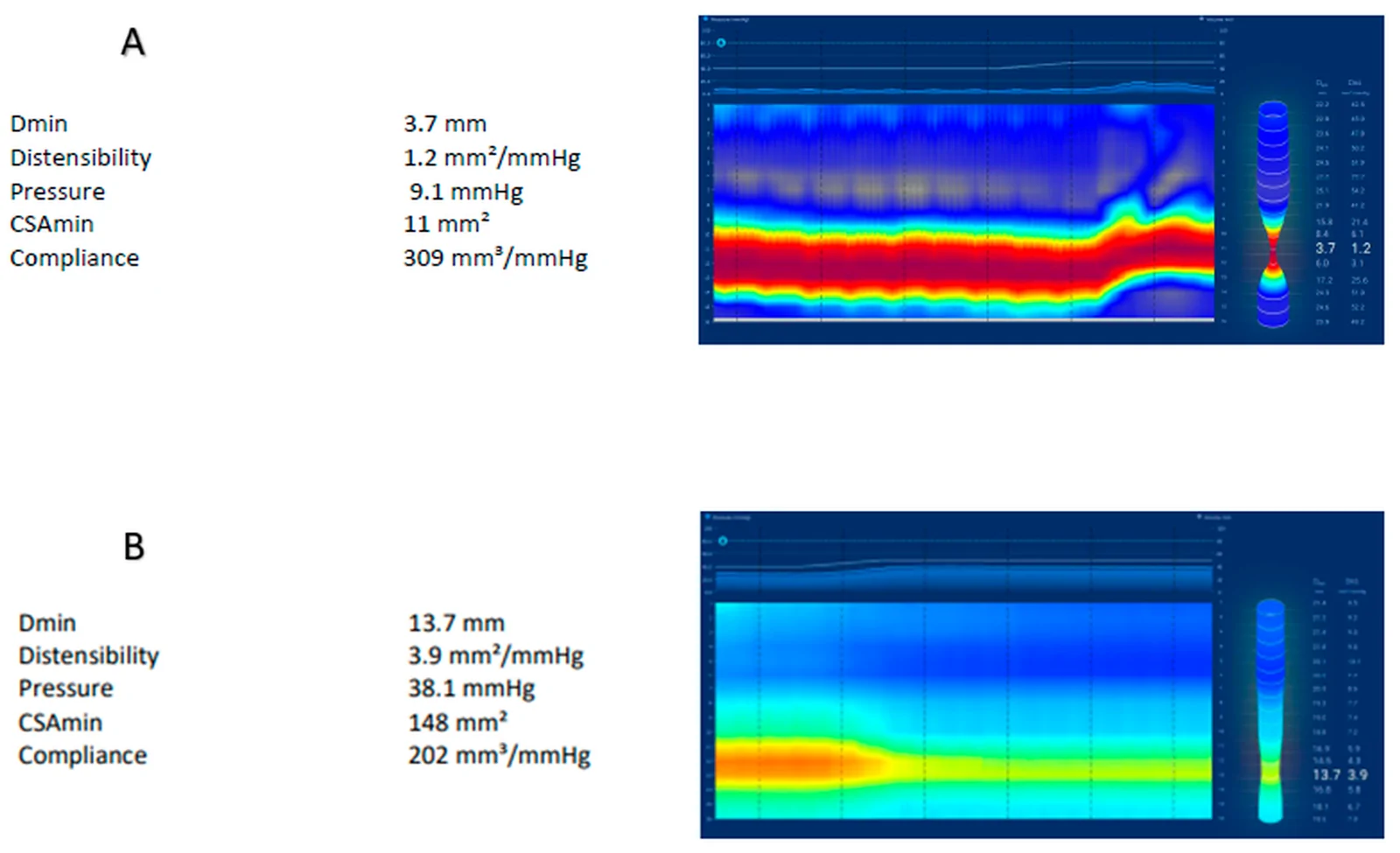
Representative EndoFLIP assessment in a patient with dysphagia and type I achalasia according to high-resolution manometry before and at the end of a peroral endoscopic myotomy (POEM). (**A**) Pre-procedural measurements (filling volume: 40 mL). (**B**) Post-procedural measurements demonstrating improved esophagogastric junction biomechanical properties following myotomy (filling volume: 40 mL). Dmin, minimum diameter; CSAmin, minimum cross-sectional area. This figure is from the corresponding author’s laboratory and illustrates an EndoFLIP assessment procedure.

Thereafter, multiple studies have tried to evaluate its use in the diagnosis of motility disorders and provide data concerning normal and diagnostic values of basic EndoFLIP metrics. In a prospective study among 20 control subjects, the authors observed that all 20 subjects had an EGJ-DI > 2.8 mm^2^/mmHg [10]. Moreover, Carlson et al. [11] evaluated 687 adult patients that completed FLIP and HRM for primary esophageal motility evaluation as well as 35 asymptomatic volunteers. All 35 controls had an EGJ-DI > 3.0 mm^2^/mmHg and a maximum EGJ diameter > 16 mm. Among the 241 patients with a reduced EGJ opening (EGJ-DI < 2.0 mm^2^/mmHg and maximum EGJ diameter < 12 mm) on FLIP planimetry, 86% had a conclusive disorder of EGJ outflow per Chicago Classification. Among the 203 patients with a normal EGJ opening (NEO: EGJ-DI ≥ 2.0 mm^2^/mmHg and maximum EGJ diameter ≥ 16 mm) on FLIP planimetry, 99% had a normal EGJ outflow per Chicago Classification [11]. Additionally, a retrospective study by Rooney et al. [12] including 240 patients with achalasia and 42 controls found that 91% of achalasia patients had an EGJ-DI ≤ 2.0 mm^2^/mmHg and 97% had an EGJ-DI ≤ 3.0 mm^2^/mmHg, while none of the controls had an EGJ-DI ≤ 2.0 mm^2^/mmHg and 3/42 (7%) had an EGJ-DI ≤ 3.0 mm^2^/mmHg. What is more, 84% of achalasia patients had a maximum EGJ-diameter of <12 mm and 231/240 (96%) had a maximum EGJ-diameter < 16 mm. All seven of the patients with an EGJ-DI > 3.0 mm^2^/mmHg had a maximum EGJ-diameter < 16 mm. All of the controls had a maximum EGJ-diameter > 16 mm [12]. The DI cut-off of 2.0 mm^2^/mmHg at 40 mL of infusion volume was also proposed the same year in a meta-analysis including 15 studies and 154 subjects [13]. Based on such findings, the Dallas Consensus proposed that cut-offs of an EGJ-DI > 3.0 mm^2^/mmHg and an EGJ-diameter > 16 mm could exclude obstructive disorders of the EGJ, and cut-offs < 2.0 mm^2^/mmHg and an EGJ-diameter < 12 mm could diagnose them [3]. Moreover, the Dallas Consensus provided analytical guidelines on how to perform EndoFLIP, proposing specific catheter types, timeframes, and ballon inflation protocols for the evaluation of an EGJ opening [3]. Table 1 provides data about DI and maximum diameter evaluation according to the Dallas Consensus. Figure 1 illustrates EndoFLIP assessment in a patient with achalasia before and after POEM.

In a following study by Ponds et al. [14], EndoFLIP was able to discriminate patients with achalasia showing previously normal integrated relaxation pressure (IRP) during HRM as these patients were found to have decreased EGJ distensibility, and they symptomatically improved after treatment for achalasia. Such a finding led experts to propose that patients with a high suspicion of achalasia in HRM showing failed esophageal motility but an IRP not surpassing the limit of 15 mmHg should undergo an EndoFLIP examination [15,16].

Additionally, EndoFLIP has also showed a discriminatory ability to detect patients with esophagogastric junction outflow obstruction (EGJOO) in HRM that could be benefited by myotomy. In a study by Triggs et al. [17] including 34 patients with EGJOO in HRM, the authors combined repetitive contraction patterns with DI in order to detect patients that could benefit from myotomy. They observed that all 18 patients with radiographic evidence of EGJOO had an EGJ-DI less than 2 mm^2^/mmHg. Furthermore, among these patients, 9 of the 18 with an EGJ-DI less than 2 mm^2^/mmHg underwent achalasia-type treatment, and 77.8% (7/9) experienced improvement in their Eckardt score. In contrast, six patients with a normal EGJ-DI (>3 mm^2^/mmHg) were managed conservatively, and all demonstrated improvement in subsequent Eckardt scores [17]. Such findings led experts in the field to suggest that in cases with suspected EGJOO, diagnosis and further intervention should not only rely on HRM, but EndoFLIP can be used in order to dictate further treatment or not [18].

### 3.2. Esophageal Body Motility Evaluation

Multiple studies have pointed out that the contraction pattern of the secondary peristalsis observed in the esophageal body, provoked by esophageal distension after EndoFLIP balloon infusion, provides crucial information and is correlated with different esophageal body motility disturbances.

In 2016, Carlson et al. [19] demonstrated in a cohort of 10 healthy controls that stepwise distension of the FLIP bag induces repetitive antegrade contractions (RACs). These findings were confirmed in a later study among 20 healthy subjects where the authors also noted that RACs were observed in all 20 subjects while in 95% of subjects, the RAC pattern persisted for ≥10 consecutive antegrade contractions [10].

Another study evaluated the response pattern among 145 patients with dysphagia submitted for EndoFLIP [20]. The authors observed that FLIP topography contractility patterns differed among HRM esophageal motility diagnoses. According to the authors, among the 111 patients with a major motility disorder on HRM, 95% exhibited a FLIP topography pattern consistent with a major motility disorder. Furthermore, all 70 patients with achalasia on HRM had abnormal FLIP topographies, while an abnormal FLIP topography was also observed in 87% of patients with EGJOO on HRM. More specifically, patients with type I achalasia exhibited absent contractility, whereas those with type III achalasia demonstrated an abnormal EGJ-DI accompanied by repetitive retrograde contractions (RRCs), consistent with a spastic pattern. In contrast, substantially greater heterogeneity in FLIP topography classifications was observed among patients with type II achalasia and EGJOO [20]. Subsequently, another prospective study including 40 patients, 80% of whom reported dysphagia, found that all nine patients later diagnosed with achalasia by HRM exhibited an abnormal FLIP response pattern, characterized by either absent contractility during distension or retrograde contractions [21]. Two years later, in 2021, once again Carlson et al. [22] provided evidence that EndoFLIP can differentiate between different achalasia subtypes. The authors analyzed the EndoFLIP data from 180 adult patients with treatment-naïve achalasia defined by HRM and concluded that proportions of patients with absent contractile responses, repetitive retrograde contractile patterns, occluding contractions, sustained occluding contractions (SOCs), and contraction-associated pressure changes >10 mmHg all differed between HRM achalasia subtypes, a fact that could help differentiate patients with spastic (type III) from non-spastic achalasia (types I and II) [22].

Accordingly, the Dallas Consensus provided further guidance on performing EndoFLIP measurements and interpreting response patterns to establish a diagnosis in patients with suspected esophageal motility disorders [3]. In addition to the above, a study combining EndoFLIP data (FLIP topography and EGJ opening parameters) with endoscopic findings, expressed through the CARS score (Contents, Anatomy, Resistance, and Stasis), provided further evidence supporting its diagnostic utility [23]. The authors found that the combination of a CARS score ≥ 4 and a FLIP planimetry classification of non-spastic obstruction identified achalasia or conclusive EGJOO in all 65 patients with such a diagnosis on HRM. Conversely, no patient with a CARS score of 0–1 and a normal EGJ opening on FLIP planimetry had achalasia or conclusive EGJOO, corresponding to a negative predictive value of 100% [23].

Nowadays, EndoFLIP use is proposed in the diagnostic algorithm of esophageal motility disorders, especially in cases where a final diagnosis with the use of HRM cannot be reached [15,16,18,24,25,26]. Nonetheless, it must be stated that EndoFLIP cannot act as a standalone examination and can act only as a complementary tool in patients with compatible symptoms after thorough evaluation via HRM, endoscopy, and barium esophagogram.

## 4. EndoFLIP During Achalasia Treatment

### 4.1. Intra-Procedural Guidance

The use of EndoFLIP during myotomy and its aid in tailoring the myotomy length and depth during the procedure in order to reach optimal procedure efficacy in terms of symptom relief and gastroesophageal reflux disease (GERD) risk minimization post-myotomy has been assessed in multiple studies.

A pivotal study published in 2013 including patients with achalasia undergoing POEM and healthy controls concluded that intraoperative EGJ profiling may be an important tool for objectively guiding the extent and completeness of the myotomy [27]. In 2015, Teitelbaum et al. [28] conducted a study involving 56 patients with achalasia who underwent myotomy (20 Heller myotomy and 36 POEM) and were evaluated with EndoFLIP at baseline after induction of anesthesia and again upon completion of the procedure (8 cm catheter, ballon inflation at 40 mL). Among patients submitted to POEM, there was no correlation between change in DI and symptoms, although patients within an “ideal” final DI range (4.5–8.5 mm^2^/mmHg) had optimal symptomatic outcomes (i.e., Eckardt ≤ 1 and Gerd Questionnaire ≤ 7) in 88% of cases, compared with 47% of those with a final DI above or below that range (*p* < 0.05) [28]. In 2020, Su et al., in a study including 77 patients with achalasia submitted to POEM or laparoscopic Heller myotomy and evaluated with EndoFLIP before and after the procedure (8 cm catheter, balloon inflated at 30 mL), presented data showing that patients with a post-operative Eckardt score ≥ 3 were significantly more likely to have a final DI ≤ 3.1 mm^2^/mmHg (*p* = 0.014) or a change in DI ≤ 3.0 mm^2^/mmHg (*p* = 0.010), while a final minimum diameter at LES > 11.0 mm was predictive of worse reflux at 2 years (*p* = 0.01) [29]. Moreover, Holstrom et al. [30], in a study including 143 patients with achalasia submitted to POEM, assessed the intraoperative use of EndoFLIP and compared it with the absence of EndoFLIP during the procedure. According to the authors, EndoFLIP application led to an additional myotomy in 65% of patients following the initial post-myotomy FLIP assessment. Moreover, at 12 months after POEM, the FLIP-guided cohort demonstrated a significantly higher clinical success rate (defined as Eckardt score ≤ 3) compared with patients in whom EndoFLIP was not used (93% vs. 81%, *p* < 0.05) [30].

On the contrary, studies do exist that do not show an additional effect in treatment efficacy when using EndoFLIP in order to tailor the extent of myotomy. Specifically, Evensen et al. [31], in a study including 62 patients with achalasia, also assessed the use of EndoFLIP during POEM. The authors concluded that intraoperative DI was not correlated with 1-year Eckardt score or DI at one-year post-myotomy. Unfortunately, there is some concern regarding how the authors defined optimal post-myotomy DI when using intraoperative EndoFLIP, as they considered the myotomy sufficient if the final DI was at least >2 mm^2^/mmHg compared to the pre-myotomy value. According to the authors’ results, the mean post-myotomy DI value in patients in whom EndoFLIP was used was 3.16 mm^2^/mmHg (2.54–4.03). Consequently, in the EndoFLIP-tailored cohort there were patients with a final DI < 3 mm^2^/mmHg, which, according to the abovementioned studies, is considered inadequate [31]. Polcz et al. [32], in a study including 34 patients with achalasia, concluded that post-myotomy EndoFLIP values were not associated with postoperative Eckardt scores or GERD. Finally, in a study by Eke et al. [33] including 168 patients submitted to POEM, with intraoperative use of EndoFLIP, the authors concluded that EndoFLIP led to myotomy extension in only 5% of patients submitted to POEM, while also showing that post-myotomy DI was increased in patients who developed GERD.

Recently, a meta-analysis including 21 studies and 1455 patients was published to evaluate whether intraoperative EndoFLIP use improves patient outcomes [34]. The authors reported that intraoperative FLIP was associated with a lower incidence of reflux esophagitis. However, this effect did not extend to clinical success or Eckardt scores. These findings suggest that the use of FLIP during myotomy may help reduce excessive dissection and subsequent GERD [34]. Based on the abovementioned data, the American Gastroenterological Association (AGA) recently suggested that EndoFLIP may be used intraoperatively to tailor the myotomy. Available evidence indicates that the final DI is correlated with the risk of post-procedural GERD. Moreover, the final DI may also be associated with myotomy effectiveness, as reflected by post-myotomy Eckardt scores, although conflicting data remain in this field [35]. Unfortunately, no standardized protocol for intraoperative EndoFLIP assessment during myotomy (e.g., catheter type and balloon filling volume) can currently be recommended, as the available evidence is derived from heterogeneous studies and cannot be readily generalized.

### 4.2. Use of EndoFLIP to Evaluate Symptom Recurrence Post-Myotomy

Several studies assessed EndoFLIP’s ability to diagnose post-myotomy clinical failure. Initially, a prospective study including 44 patients submitted either to Heller myotomy or POEM or balloon dilatation showed that a DI cut-off of <2.8 mm^2^/mmHg (balloon dilated at 60 mL) provides a diagnosis treatment failure sensitivity of 77.3% [36]. DeWitt et al. [37] also used the same cut-off in order to document post-myotomy clinical response, and a sensitivity of 91.8% was observed. Specific analysis utilizing area under the curve, in order to evaluate EndoFLIP predictive value in symptom recurrence among patients with achalasia submitted to POEM, was provided in a recent study by Yoo et al. [38]. According to the researchers, patients with a post-POEM DI below 7 mm^2^/mmHg (30 or 40 mL) had a significantly higher rate of incomplete response after POEM (*p* = 0.001). Specifically, they showed that a DI of 6.6 mm^2^/mmHg at a post-DI of 30 mL (area under the ROC: 0.66) and 9.9 mm^2^/mmHg at a post-DI of 40 mL (area under the ROC: 0.72) could predict treatment failure [38]. The moderate ability of EndoFLIP in documenting clinical failure post-myotomy was also presented in a recent prospective study, in which the measured AUC of DI was 0.72 (0.53–0.90), while no optimal DI cut-off was proposed [39].

In another study by Kamboj et al. [40] including 222 patients with prior achalasia treatment, the authors observed that only the change in DI and diameter on EndoFLIP was associated with clinical response based on an Eckardt score ≤ 3, but not post-POEM DI and EGJ diameter values. Finally, a recent study by Ichkhanian et al. reported that the combination of DI and IRP, measured by HRM, could provide the greatest diagnostic accuracy when compared with isolated use of either IRP or DI [41].

When considering all available evidence, no specific DI threshold has been shown to accurately identify patients with post-myotomy treatment failure who are likely to benefit from further intervention, while other EndoFLIP metrics have not been thoroughly evaluated in such a setting. Until newer data become available, EndoFLIP may serve as a complementary tool to clinical manifestations, barium esophagogram, and HRM. However, its results should be interpreted with caution in patients with recurrent symptoms following myotomy for achalasia. Additionally, no standardized protocol for EndoFLIP performance can be proposed in such a setting (catheter type, balloon filing volume, etc.) as data are based on heterogenous studies and cannot be generalized. It must be stated that in most studies, a 16 cm catheter was used—but not unanimously [41]—and DI was assessed when using a balloon inflation volume of 60 mL [39,40], though unfortunately not consistently across all studies [38,41].

## 5. GERD and Fundoplication

A handful of studies have evaluated the role of EndoFLIP in the assessment of the EGJ in GERD. Initial studies revealed that the EGJ-DI was significantly higher in patients with GERD than in controls (*p* = 0.04) [42]. However, subsequent studies failed to demonstrate an association between DI values and either acid exposure time or clinical outcomes [43,44]. In contrast, EndoFLIP has been more extensively utilized to assess EGJ alterations following fundoplication. The ultimate goal of surgical treatment is to restore the function of the anti-reflux barrier at the EGJ by creating a new valve. Optimal outcomes depend on achieving an appropriate balance in the tightness of the new valve. An overly tight valve may predispose patients to dysphagia and gas-bloat syndrome, whereas an overly loose valve may increase the risk of recurrence of GERD symptoms.

In this setting, intraoperative EndoFLIP measurements may provide valuable information and serve as an objective guide for surgeons. Recently, several studies have assessed its role in this indication; however, results have varied. This variability likely reflects significant differences in both EndoFLIP-related factors (e.g., balloon size and inflation volume) and procedural variables across the literature. In particular, assessment timing, the use of pneumoperitoneum, and the type of fundoplication (e.g., Nissen or Toupet) have all been shown to influence measurement outcomes [45]. Su et al. [46], in a cohort of 175 patients undergoing fundoplication, reported that a post-procedural DI < 2.0 mm^2^/mmHg at 40 mL was significantly associated with the presence of gas-bloat syndrome and dysphagia at 1-year follow-up (*p* = 0.040 and *p* = 0.025, respectively). Moreover, they proposed an optimal DI range of 2.0–3.5 mm^2^/mmHg for overall symptom control. However, other studies have found contradictory results [47,48,49]. For example, Sabour et al. [47] recently suggested that a post-procedural DI > 0.5 mm^2^/mmHg measured with an 8 cm catheter at 40 mL is adequate to minimize the risk of dysphagia. Additionally, an upper limit of <4.5 mm^2^/mmHg was identified to reduce the risk of GERD symptom recurrence. Similarly, another retrospective study of 163 patients, with a post-fundoplication DI > 0.5 mm^2^/mmHg (8 cm catheter, 40 mL fill volume), reported that only one patient required endoscopic dilation for dysphagia at 3 months [48]. Conversely, two earlier studies reported no correlation between post-operative DI measurements and clinical outcomes [50,51].

To address the high variability in DI values, Wu et al. [52] evaluated whether compliance could serve as a complementary predictor of outcomes, given its more global assessment of EGJ mechanical properties. They identified a significant association between post-procedural compliance and DI, with compliance values between 80 and 92 mm^3^/mmHg associated with the most favorable outcomes. Unfortunately, once again, no standardized protocol for EndoFLIP performance can be proposed (catheter type, balloon filing volume, etc.) as data are based on heterogenous studies and cannot be generalized.

## 6. Eosinophilic Esophagitis (EoE)

EoE is a chronic, immune-driven esophageal disorder characterized predominantly by dysphagia, reflecting progressive fibrostenotic remodeling of the esophageal wall. This remodeling leads to the formation of strictures, which in turn predispose patients to recurrent food impaction events. Endoscopy remains the cornerstone for assessing and monitoring disease activity, providing direct visualization of mucosal abnormalities while enabling histologic evaluation through biopsies. However, histologic assessment based on eosinophil counts often shows poor correlation with fibrotic remodeling, while endoscopic evaluation is limited by issues of reproducibility and interobserver variability. Defining the fibrostenotic phenotype early in the course of EoE is pivotal for recognizing patients at increased risk of complications requiring endoscopic dilation.

Kwiatek et al. [53] first applied EndoFLIP to evaluate esophageal distensibility in 35 patients with EoE and 15 healthy controls, demonstrating that the DI was significantly lower in the patient group (*p* = 0.02). Another prospective study of 70 patients found that a reduced distensibility plateau (<225 mm^2^) was associated with food impaction, both at baseline and during follow-up [54]. Moreover, patients requiring dilation had a significantly lower distensibility plateau compared with those not requiring dilation. Of note, no correlation was found between the degree of esophageal eosinophilia and clinical outcomes. Furthermore, data suggest an association between EndoFLIP measurements and endoscopic features of fibrostenosis in both adult and pediatric patients. Chen et al. [55], in a prospective cohort of 72 patients with EoE, reported that a decreased distensibility plateau was associated with higher ring severity according to the Endoscopic Reference Score (EREFS) (*p* < 0.001). Similarly, a prospective study consisting of 59 pediatric patients demonstrated that those with fibrostenotic endoscopic features had a significantly lower DI compared with those presenting normal (mean, 3.7 ± 1.8 vs. 6.6 ± 1.8; *p* = 0.0003) or predominantly inflammatory findings (mean, 3.7 ± 1.8 vs. 5.1 ± 1.5; *p* = 0.02) [56]. Finally, Almazan et al. [57] in a retrospective study reported that the DI was linked with histological markers of remodeling, such as dilated intercellular spaces and basal zone hyperplasia (*p* < 0.05). In accordance with previous studies, no significant relationship was observed between the DI and eosinophilic count (*p* = 0.56).

Taken together, the available evidence suggests that EndoFLIP measurements reliably reflect fibrostenotic features when present. In fact, AGA guidelines recognize it as a valuable diagnostic tool, especially when endoscopic and/or histologic findings are discordant with clinical symptoms [24]. Unfortunately, no standardized protocol for EndoFLIP performance can be proposed (catheter type, balloon filing volume, etc.) as data are based on heterogenous studies and cannot be generalized. Compared with endoscopic assessment, a key advantage of EndoFLIP is its more objective nature. In this context, some authors propose that it may be used alongside eosinophil counts to assess the remodeling process in the course of EoE.

## 7. Pyloric Assessment in Gastroparesis

### 7.1. Pre-Treatment Assessment

Gourcerol et al. [58] first reported the use of EndoFLIP for pyloric sphincter assessment in a cohort of 53 individuals. They found that mean pyloric compliance at a 40 mL distension volume was significantly lower in patients with gastroparesis compared to healthy volunteers (16.9 ± 2.1 vs. 25 ± 2.4 mm^2^/mmHg, respectively; *p* < 0.05), and was correlated with both symptom severity and gastric emptying [58]. Moreover, using the 90th percentile of the healthy group, they defined a compliance cut-off of 10 mm^2^/mmHg as the lower limit of normal. Snape et al. [59], among 114 patients with nausea and vomiting, also found that patients with gastroparesis had altered pyloric parameters compared to patients with normal gastric emptying. Pyloric distensibility was also significantly lower in the former group (8.0 ± 1.0 vs. 12.5 ± 1.4 mm^2^/mmHg in the latter; *p* < 0.01). Subsequently, several studies assessed whether pre-procedural EndoFLIP abnormalities could predict clinical success, identifying patients most likely to benefit from pyloric procedures. In the previously described study, Gourcerol et al. [58] reported that pyloric hydraulic dilation improved both gastric emptying and symptoms in 10 patients with low pyloric compliance.

**Table 1:** Indications of EndoFLIP, topographic assessment, and major metrics assessed—cut-offs proposed by Dallas Consensus and exploratory cut-offs.

	Assessment	Most Common Metrics and Cut-Offs Used
**Esophageal motility disorder diagnosis ^#^**	EGJ evaluation [3] (Balloon filling volume for DI: 60 mL, and for maximum EGJ diameter: 70 mL) Esophageal body motility evaluation [3]	Abnormal: DI < 2.0 mm^2^/mmHg and maximum EGJ diameter < 12 mm Normal: DI > 3 mm^2^/mmHg and maximum EGJ diameter > 16 mm Inconclusive EGJ opening: DI < 2 mm^2^/mmHg or maximum EGJ diameter < 16 mm but no reduced EGJ opening **Normal** Antegrade contractions **Abnormal** Repetitive retrograde/absent contractions SOC (a non-propagating retrograde or horizontal contraction in the body persisting for >10 s) sLESC (transient reduction in LES diameter lasting >5 s with associated increase in FLIP pressure
**Intraprocedural use during myotomy for achalasia**	EGJ evaluation [28,29]	Eckardt score, GERD Final DI > 3 mm^2^/mmHg * and <8.5 mm^2^/mmHg *
**Evaluation of symptom recurrence post-myotomy**	EGJ evaluation [36,37]	Final DI > 2.8 mm^2^/mmHg ^#^
**GERD Fundoplication**	EGJ evaluation [47,48,49]	DI > 0.5 mm^2^/mmHg (8 cm catheter, 40 mL fill volume): avoidance of post-procedural dysphagia * DI < 4.5 mm^2^/mmHg (8 cm catheter, 40 mL fill volume): avoidance of symptom recurrence *
**EoE**	Esophageal body evaluation	No proposed threshold
**Gastroparesis** **pre-treatment assessment**	Pyloric sphincter evaluation [60,61,62]	DI < 10 mm^2^/mmHg (8 cm catheter, 40 mL fill volume); DI < 9.2 mm^2^/mmHg (8 cm catheter, 50 mL fill volume): predicts response to pyloric interventions *
**Gastroparesis** **post-treatment assessment**	Pyloric sphincter evaluation	No proposed threshold

* Not consensus-supported, based on limited studies; not well-defined cut-offs, EndoFLIP performance protocol not standardized in such settings. ^#^ Consensus supported. ^#^ Poor predictive ability. EGJ: Esophagogastric Junction, DI: Distensibility Index, GERD: Gastroesophageal Reflux Disease, EoE: Eosinophilic Esophagitis, SOC: Sustained Occluding Contraction, sLESC: Sustained Lower Esophageal Sphincter Contraction.

Desprez et al. [60] used the cut-off of 10 mm^2^/mmHg at 40 mL using an 8 cm catheter to stratify patients with refractory gastroparesis undergoing botulinum toxin injections into those with altered distensibility (*n* = 19) and those with normal distensibility (*n* = 16). Both total symptom severity and gastric emptying time were significantly reduced in the former group at 3 months, whereas no significant change was observed in the latter (*p* < 0.01). Regarding individual symptoms, early satiety and bloating were the most improved in patients with altered pyloric distensibility. Jacques et al. [61] reported in a prospective cohort of 20 patients undergoing gastric peroral endoscopic myotomy (G-POEM) that a pre-procedural distensibility threshold of <9.2 mm^2^/mmHg at 50 mL yielded 72.2% sensitivity and 100% specificity for predicting clinical success. Accordingly, this cut-off demonstrated excellent performance in identifying patients who would benefit from G-POEM; however, this was at the expense of reduced ability to exclude success in patients with higher values. A more recent prospective study reported that mean distensibility prior to G-POEM was significantly lower in clinical responders at 6 months compared to non-responders (7.10 vs. 9.24 mm^2^/mmHg; *p* = 0.002) [62]. A specific DI cut-off of 7.35 mm^2^/mmHg was deemed the best predictor of clinical success, with a specificity of 80.8% and a sensitivity of 60.6%.

Despite most studies providing evidence of an inverse relationship between baseline pyloric distensibility and diameter and post-intervention clinical efficacy, two studies have reported contradictory results. For example, a retrospective study demonstrated that patients with clinical response at 1-year post-G-POEM had significantly higher baseline pyloric distensibility (9.9 ± 6.5 vs. 5.6 ± 2.4 mm^2^/mmHg; *p* < 0.05) and CSA (196.1 ± 74.7 vs. 137.2 ± 64.4 mm^2^; *p* < 0.05) compared to non-responders [63]. In this cohort, patients with low pyloric compliance and no improvement after G-POEM may represent a distinct phenotype with limited potential to benefit from pyloric interventions, likely reflecting severely impaired pyloric function. Once more though, no standardized protocol for EndoFLIP performance can be proposed in such a setting (catheter type, balloon filing volume, etc.) as data are based on heterogenous studies and cannot be generalized. Major studies reporting EndoFLIP assessment of the pylorus in patients with gastroparesis are presented in Table 2.

### 7.2. Post-Treatment Assessment

In addition to patient selection, EndoFLIP can also serve as a tool to evaluate the technical efficacy of pyloric interventions by assessing improvements in pyloric parameters post-procedurally. Moreover, a handful of studies have also aimed to determine whether these improvements correlate with clinical outcomes. In a retrospective study of 37 patients with refractory gastroparesis treated with G-POEM, both the CSA and DI at 40 mL and 50 mL were significantly increased after the procedure. Moreover, post-procedural measurements were significantly better in clinical responders at 1 year compared to non-responders (*p* < 0.05) [64]. In regression analysis, the CSA following intervention emerged as the best predictor of clinical efficacy (*p* = 0.08), with cut-offs of 154 mm^2^ at 40 mL and 247 mm^2^ at 50 mL showing the strongest association. Similarly, Conchillo et al. [64], in a prospective study of 24 patients who underwent G-POEM, found that clinical response at 6 months was significantly associated with an increase in pyloric distensibility measured at 3 months post-procedure (χ^2^ = 8.6, *p* = 0.003). In contrast to the study by Vousoughi et al. [63], no pyloric measurements were found to predict clinical success. Moreover, Martinek et al. [67], in the only randomized trial evaluating G-POEM, reported that a DI > 13 mm^2^/mmHg after the procedure could predict clinical success; however, this was not statistically significant. Murray et al. [66] treated 46 patients with refractory gastroparesis using the esophageal Functional Lumen Imaging Probe (EsoFLIP) dilation system combined with EndoFLIP assessment before and after the procedure. Consistent with previous studies, the authors found a significant correlation between improvements in the DI and clinical response.

Additionally, Jehangir et al. [65] proposed a novel role for EndoFLIP as a tool to guide management in patients with an inadequate response to pyloric interventions. Currently, there is a lack of data regarding whether an additional pyloric intervention or an alternative treatment method is appropriate for this group of patients. In a small cohort of 13 patients with refractory symptoms despite prior pyloromyotomy or pyloroplasty, those who responded to a subsequent balloon dilatation had lower baseline pyloric distensibility compared to non-responders. According to the authors, these patients may have had an incomplete pyloric response to the preceding interventions and thus required additional treatment. Nevertheless, no measurements prior to the initial procedures were carried out to further support this hypothesis.

Overall, EndoFLIP assessment appears to reflect the physiological response of the pylorus after the procedure, which is associated with clinical outcomes. However, current data remain limited regarding which parameter provides the greatest predictive value. More importantly, defining standardized cut-offs is strongly needed to guide procedural adequacy and help endoscopists optimize the likelihood of clinical success.

## 8. Clinical Implications and Future Directions

Table 2 summarizes the major indications for EndoFLIP assessment, along with the corresponding metrics and proposed cut-offs. When it comes to esophageal motility disorders, and especially disorders which are mainly driven by obstruction in the level of the LES, EndoFLIP can act complementary to HRM and, in ambiguous cases, confirm the final diagnosis in patients with a compatible clinical presentation when taken together with data from endoscopy and barium esophagogram. What is more, data have provided evidence that EndoFLIP can also help in the diagnosis of esophageal motility disorders, especially spastic disorders. Additionally, its use has expanded in tailoring myotomy, as it is now well-documented that it can help towards minimization of post-myotomy GERD development and, possibly (although data are conflicting), increased efficacy of the intervention. Unfortunately, its role in predicting which patients with recurrent symptoms following myotomy are likely to benefit from further intervention remains unclear. EndoFLIP may still serve as a complementary tool in this setting, although its findings should be interpreted in the context of clinical symptoms and other diagnostic modalities, including endoscopy, barium esophagography, and HRM.

Although commonly used sedative agents appear to have no significant impact on EndoFLIP measurements, anticholinergic agents may influence them by blocking acetylcholine-mediated smooth muscle contraction, resulting in reduced gastrointestinal sphincter tone and increased distensibility. However, evidence from clinical studies remains scarce. A prospective study in healthy volunteers demonstrated that intravenous hyoscine butylbromide significantly increased pyloric distensibility, as measured by EndoFLIP [68].

Several studies have demonstrated that altered pyloric characteristics, particularly a reduced DI at baseline, are strongly associated with clinical response following pyloric interventions, mainly G-POEM. A lower DI likely reflects pylorospasm and impaired pyloric distensibility. However, extremely low values may indicate advanced disease, in which pyloric interventions are less likely to be effective. Current data suggest that a pre-procedural DI < 9–10 mm^2^/mmHg is predictive of response to pyloric interventions. Moreover, the association between post-procedural parameters and clinical outcomes provides a rationale for using EndoFLIP to guide interventions by targeting predefined values; however, data supporting this approach remain limited [69]. The timing of post-procedural assessment currently varies across studies, ranging from immediately after the intervention to 3 months later. The rationale for a delayed assessment is to minimize the potential impact of procedure-related inflammation, which may influence EndoFLIP measurements. Conversely, repeat EndoFLIP measurement at 3 months requires repeat sedation, with its associated risks. Currently, only Vousoughi et al. [63] compared these two approaches and found that measurements obtained immediately after the intervention were a better outcome predictor than those obtained 3 months later.

Overall, current data suggest that EndoFLIP may represent a valuable tool for both predicting fundoplication outcomes and assessing fibrostenotic changes in EoE. During fundoplication, avoiding extreme post-operative DI values—for example, no lower than 0.5 mm^2^/mmHg—may be reasonable [47].

Despite the growing evidence supporting the use of EndoFLIP, there is still room for improving the standardization of the technique. Since EndoFLIP measurements are influenced by several factors, such as catheter type, filling volume, and timing of assessment, establishing standardized protocols would help clinicians more accurately interpret the results. Moreover, further studies, especially prospective ones, are required to better define optimal cut-offs and improve clinical decision-making. This is particularly important for pyloric assessment in gastroparesis, as well as for fundoplication and EoE, where the available evidence remains more limited than in achalasia. Well-validated cut-offs may further support the implementation of EndoFLIP-guided interventions in clinical practice. Besides technical optimization and further clinical validation, another important area for future research is to better define the role of EndoFLIP alongside other diagnostic modalities, including HRM, endoscopy, timed barium esophagography, and clinical assessment, across different indications. Until more evidence becomes available, EndoFLIP should be considered a complementary rather than a standalone diagnostic tool. Finally, the integration of artificial intelligence (AI)-based FLIP topography interpretation has the potential to improve pattern recognition, reduce interobserver variability, and facilitate wider clinical adoption.

## 9. Conclusions

EndoFLIP has emerged as a useful complementary tool for the assessment of upper gastrointestinal biomechanics. Its most established role is in the evaluation of the EGJ opening and distension-induced contractility in patients with suspected esophageal motility disorders. Increasing evidence also supports its use in procedural guidance and in the assessment of fibrostenotic remodeling in eosinophilic esophagitis and pyloric dysfunction in gastroparesis, although further evidence is needed until its use can be supported outside a research setting. Nevertheless, its broader clinical adoption remains limited by heterogeneous protocols, inconsistent thresholds, and a predominance of observational evidence. Prospective studies using standardized measurement protocols are needed to validate clinically relevant thresholds and determine whether EndoFLIP-guided management improves patient outcomes.

## Figures and Tables

**Table 2:** Summary of major studies evaluating EndoFLIP for pyloric assessment in gastroparesis.

Author and Year	Procedure	Study Design	Number of Patients	Assessment Timing	EndoFLIP Measurement	Findings
Jacques et al. [61] (2019)	G-POEM	Single-center prospective	20	Pre/Post (3 months)	DI (mm^2^/mmHg)	Baseline DI < 9.2 mm^2^/mmHg predicted clinical response with 100% specificity and 72.2% sensitivity (*p* = 0.04).
Vosoughi et al. [63] (2020)	G-POEM	Multicenter retrospective	37	Pre/Post (Immediately or 3 months)	CSA (mm^2^) DI (mm^2^/mmHg)	Pre: Increased DI at 40 mL and CSA at 50 mL correlated with clinical improvement (*p* < 0.05). Post: Improvements in CSA and DI were linked to clinical response at 1 year; a CSA threshold > 154 mm^2^ at 40 mL demonstrated 77% accuracy (71% sensitivity, 91% specificity) for predicting success.
Conchillo et al. [64] (2021)	G-POEM	Single-center prospective	24	Post (3 months)	DI (mm^2^/mmHg)	Improvement in DI correlated with clinical response at 6 months (χ^2^ = 8.6, *p* = 0.003).
Desprez et al. [60] (2019)	Botulinum toxin injection	Multicenter prospective	35	Pre	DI (mm^2^/mmHg)	A baseline DI < 10 mm^2^/mmHg was predictive of symptomatic improvement at 3 months.
Jehangir et al. [65] (2021)	Pyloric balloon dilation	Single-center retrospective	13	Pre	DI (mm^2^/mmHg)	Lower baseline distensibility was observed among patients who showed symptomatic improvement compared with those without clinical response (40 mL: 7.2 ± 1.0 vs. 13.9 ± 2.1 mm^2^/mmHg, *p* = 0.02).
Murray et al. [66] (2021)	Pyloric balloon dilation	Single-center retrospective	46	Post	DI (mm^2^/mmHg)	Each 10 mm^2^/mmHg increase in post-interventional DI was associated with a 0.9-point reduction in GSCI (*p* = 0.012).
Farooq et al. [62] (2025)	G-POEM	Single-center prospective	90	Pre	DI (mm^2^/mmHg)	Baseline DI was significantly lower in responders compared with non-responders (7.10 ± 2.75 vs. 9.24 ± 3.14 mm^2^/mmHg; *p* = 0.002). A pre-interventional DI threshold of 7.35 mm^2^/mmHg predicted response with an AUC of 0.72, 80.8% specificity, and 60.6% sensitivity.

EndoFLIP: Endoscopic Functional Luminal Imaging Probe, G-POEM: Gastric Peroral Endoscopic Pyloromyotomy, DI: Distensibility Index, CSA: Cross-Sectional Area.

## Data Availability

No new data were created or analyzed in this study. Data sharing is not applicable to this article.
